# Mechanistic insights into the synaptic damage-repair and regeneration processes in neurodegenerative Alzheimer’s disease: phytochemicals as neuroprotective agents

**DOI:** 10.3389/fnsyn.2026.1835778

**Published:** 2026-06-11

**Authors:** Bharti Yadav, Prabhakar Gangwar, Kirti Kumari, Anuradha Yadav, Rashmi Rao, Keshav Kumar, Harkomal Verma, Monisha Dhiman, Anil Kumar Mantha

**Affiliations:** 1 Department of Zoology, School of Basic Sciences, Central University of Punjab, Bathinda, India; 2 Department of Microbiology, School of Basic Sciences, Central University of Punjab, Bathinda, India

**Keywords:** Alzheimer’s disease, cytoskeleton, exosomes, mitochondrial dysfunction, NETosis, phytochemicals, synaptic plasticity

## Abstract

Synaptic failure is one of the earliest and most significant contributors to the cognitive decline in Alzheimer’s disease (AD), preceding extensive neuronal loss. Although amyloid beta (Aβ) plaques and neurofibrillary tangles (NFTs) of tau protein characterize the disease, memory impairment primarily results from the gradual deterioration of synaptic communications. This decline is caused by a complex interaction among mitochondrial energy deficits, cytoskeletal instability, disrupted exosomal signaling, and immune-mediated synaptic pruning. Mitochondrial dysfunction, particularly affecting complexes I and IV, leads to reduced ATP production, faulty mitophagy and disrupted calcium (Ca^2+^) homeostasis, placing the synapse under constant metabolic stress. Elevated reactive oxygen species (ROS) further activate stress pathways, including p38 MAPK and JNK, contributing to synaptic protein damage and impaired long-term potentiation (LTP). Furthermore, tau hyperphosphorylation destabilizes the neuronal cytoskeleton, weakening dendritic spine integrity and synaptic connectivity. At the same time, Aβ alters the cargo carried by exosomes, facilitating the spread of pathogenic Aβ and tau species between the neurons and modulating microglial activation and complement-mediated synaptic pruning. Additionally, emerging studies highlight the role of NETosis in exacerbating neuroinflammation and compromising the blood-brain barrier (BBB) integrity, thereby increasing synaptic damage. In contrast, phytochemicals such as resveratrol, ginkgolide B, curcumin, ferulic acid, epigallocatechin gallate (EGCG), and quercetin exert neuroprotection by restoring redox balance, altering exosomal communications, stabilizing cytoskeletal signaling, and reducing neuroinflammation. Moreover, delivery techniques such as nanoparticles and engineered exosomes enhance BBB permeability and enable targeted synaptic intervention. Overall, this review summarizes current mechanistic findings and highlights the potential of phytochemicals as multitarget therapeutic agents for synaptic repair and functional recovery in AD.

## 1 Introduction

Alzheimer’s disease (AD) has traditionally been defined by progressive neuronal loss and the accumulation of amyloid beta (Aβ) plaques (Sultana and Butterfield, 2010; Ballard et al., 2011). For many years, therapeutic strategies are largely directed toward reducing plaque burden or preventing widespread neurodegeneration. However, this perspective has gradually shifted, as several studies now indicate that synapse loss shows a much stronger association with the cognitive decline than plaque deposition alone. Even though individuals with substantial Aβ accumulation do not show clear cognitive symptoms, loss of synaptic density is more consistently linked with impairment in learning and memory (Aizenstein et al., 2008). These observations highlight the importance of focusing on synapses as the central sites of early dysfunction in AD.

Synapses are highly specialized and metabolically active structures that support neurotransmission, plasticity, and information processing. Their functions depend on a tightly regulated balance between redox signaling, mitochondrial bioenergetics, cytoskeletal framework, and calcium (Ca^2+^) homeostasis (Tang et al., 2019). The failure of these pathways in AD is interdependent, with dysfunction in one pathway influencing the others, creating a cascade of events that progressively destabilize synaptic function. Mitochondrial dysfunction plays a central role in this process, particularly through impairments in respiratory chain complexes and reduced ATP production. At the same time, excessive reactive oxygen species (ROS) production exceeds the capacity of endogenous antioxidant systems, leading to oxidative damage of mitochondrial DNA, lipids, as well as synaptic proteins (Tönnies and Trushina, 2017; Grimm and Eckert, 2017). This imbalance is especially critical at synapses, where energy demand is high and the ability to locally regulate Ca^2+^ and oxidative stress is limited.

On the other hand, cytoskeletal integrity and organization are significantly affected. Hyperphosphorylated tau induces microtubule destabilization, and interferes with axonal transport of synaptic vesicles and mitochondria (Alonso et al., 1994; Wu et al., 2021). As a result, synaptic terminals are deprived of essential components required for sustained activity. This ultimately leads to the collapse of dendritic spines and weakening of synaptic connections (Penzes et al., 2011). Alongside these changes, neuroinflammatory responses become more prominent. The release of inflammatory mediators and proteolytic enzymes by activated microglia further exacerbates oxidative stress and synaptic pruning, while the formation of neutrophil extracellular traps (NETosis) worsens the situation (Pietronigro et al., 2017), as reviewed by our group recently (Rao R. et al., 2026). These processes are closely interconnected, in which oxidative stress, inflammation, and metabolic dysfunction reinforce one another and gradually reduce the capacity for synaptic repair.

Despite these pathological changes, the brain retains adaptive mechanisms that help maintain synaptic stability. Synaptic plasticity enable neurons to adjust their structural and functional properties in response to activity, supporting learning and memory. Such flexibility requires regulated redox signaling, selective protein synthesis and mitochondrial bioenergetics. In addition, synaptic repair and regeneration rely on dynamic actin-driven spine remodeling and microtubule-dependent cargo transport, which ensure the precise delivery of mitochondria, receptors, and synaptic vesicles required for functional recovery (Mattson, 2007; Faria-Pereira and Morais, 2022). However, this adaptability relies on coordinated intracellular and intercellular signaling. In this context, exosomes have emerged as important mediators of communication between neurons and glial cells (Basso and Bonetto, 2016; Kaur et al., 2021). They transport lipids, proteins and regulatory RNAs that influence immune responses, cellular metabolism and synaptic remodeling. Under physiological conditions, these pathways enable network maintenance and repair (Kumar et al., 2024). However, under pathological conditions, exosomes may facilitate the spread of toxic proteins such as Aβ and tau, highlighting their dual role as both mediators of neuroprotection and contributors to AD progression.

Although individual aspects of synaptic dysfunction in AD have been widely studied, a clear understanding of how mitochondrial dysfunction, oxidative stress, neuroinflammation and cytoskeletal disruption interact at the synaptic level is still developing. This also reflects a limitation of current therapeutic strategies, as targeting a single pathway has shown limited success in restoring synaptic function. In this context, phytochemicals have gained attention due to their ability to influence multiple pathways simultaneously. For example, curcumin, through activation of the Nrf-2 signaling pathway, quercetin via modulation of NETosis, and resveratrol by promoting mitochondrial biogenesis have demonstrated multitargeted effects on oxidative stress, neuroinflammation, and synaptic function (Kaur et al., 2017; Naoi et al., 2019; Abbas et al., 2025). Along with their antioxidant properties, they can modulate mitochondrial function, enhance endogenous defense systems, regulate inflammatory pathways, and support cellular signaling involved in synaptic maintenance. When combined with modern delivery systems, including nanoparticles and engineered exosomes, these compounds may facilitate more efficient transport across the BBB and enable better targeting of the brain and synapses (Grabrucker et al., 2016; Jiang et al., 2022), as further discussed in our previous work (Kaur et al., 2017, 2021; Verma et al., 2024a).

Thus, in this review, we explore a synapse-centered mechanistic perspective on how mitochondrial dysfunction, cytoskeletal alterations, dysregulated exosomal signaling, and NETosis, together with neuroinflammation, compromise synaptic integrity in AD. We further investigate whether phytochemical interventions restore bioenergetics and redox homeostasis and examine the possible physiological mechanisms underlying synaptic resilience and functional recovery in AD.

### 1.1 Literature search strategy

A systematic literature search was conducted using PubMed, Google Scholar, and Scopus databases, covering publications from January 2010 to March 2026. Seminal and foundational studies published prior to this period were also included, where relevant, to provide appropriate mechanistic context. The following Medical Subject Headings (MeSH) terms and keyword combinations were used: “Alzheimer’s disease,” “synaptic dysfunction,” “mitochondrial dysfunction,” “tau hyperphosphorylation,” “neuroinflammation,” “NETosis,” “exosomes,” and “phytochemicals.” Studies were included if they met the following criteria: (i) peer-reviewed original research articles, systematic reviews, or meta-analyses published in English; (ii) studies directly addressing synaptic mechanisms, neuroinflammation, mitochondrial dysfunction, cytoskeletal remodeling, or exosomal signaling in AD or relevant neurodegenerative disease models; and (iii) studies reporting mechanistic, biochemical, electrophysiological, or clinical data relevant to the pathways reviewed. Studies were excluded if they: (i) lacked mechanistic data; (ii) published as abstracts, case reports, or non-peer-reviewed commentaries; and (iii) not available in the English language.

## 2 Mitochondrial bioenergetics and synaptic dysfunction in AD

Mitochondria are double-membrane-bound organelles that serve as the primary site of cellular energy production, generating ATP through oxidative phosphorylation (OXPHOS). This process, however, also leads to the generation of ROS as unavoidable byproducts of mitochondrial activity (Turrens, 2003; Oyewole and Birch-Machin, 2015). In AD, mitochondrial function is significantly impaired, with impaired OXPHOS, reduced ATP production, increased ROS levels, and disruptions in mitochondrial biogenesis and intracellular trafficking. These changes are particularly evident in metabolically active brain regions such as the cortex and hippocampus, where mitochondrial respiration, membrane potential, and cytochrome c oxidase activity are significantly affected. Given the exceptionally constant and very high supply of energy at synaptic terminals, any disruption in mitochondrial function makes them particularly vulnerable to bioenergetics failure and dysfunction (Swerdlow, 2018).

ATP generated by mitochondria plays a key role in maintaining synaptic function. It maintains the ion gradients across the neuronal membrane and promotes processes such as the mobilization of synaptic vesicles, neurotransmitter release, and synaptic plasticity (Attwell and Laughlin, 2001; Verstreken et al., 2005). Both presynaptic and postsynaptic compartments require a steady supply of ATP, primarily because maintaining Ca^2 +^ homeostasis during neurotransmission is energy demanding. Since many of these processes occur at synaptic sites, mitochondria are often located close to synapses, where they provide ATP and help regulate intracellular Ca^2 +^ levels (Li et al., 2004; Kang et al., 2008). In an AD mouse model, synaptic mitochondria tend to accumulate Aβ in an age-dependent manner and show increased oxidative stress along with reduced bioenergetic efficiency, which collectively affect synaptic activity (Du et al., 2010).

Mitochondria are involved in multiple stages of neurotransmission, including neurotransmitter synthesis, vesicle trafficking, release, and recycling through ROS regulation and Ca^2 +^ homeostasis maintenance (Vos et al., 2010; Jeanneteau and Arango-Lievano, 2016). Compromise of these functions in AD brains, evidenced by consistent OXPHOS deficits and reduced ATP levels, is closely associated with progressive synaptic dysfunction (Shulman et al., 2004; Sultana and Butterfield, 2010). Oxidative stress associated with mitochondrial dysfunction can alter synaptic membranes by peroxidizing membrane lipids, thereby interfering with fusion pore opening and affecting synaptic vesicle release, leading to vesicle accumulation at presynaptic terminals (Arai et al., 2011). Oxidative stress also promotes tau hyperphosphorylation by inhibiting phosphatases such as protein phosphatase 1 (PP1) and protein phosphatase 2A (PP2A), further disrupting synaptic vesicle trafficking (Kamat et al., 2016). Tau hyperphosphorylation, primarily mediated by kinases GSK-3β and CDK5, causes tau to dissociate from the microtubules, leading to microtubule destabilization and impaired axonal transport of synaptic vesicles and mitochondria to synaptic terminals. This energy and cargo deprivation directly amplifies synaptic dysfunction in AD (Iqbal et al., 2010; Yang et al., 2024). Since mitochondria are a major source of neuronal ROS, targeting mitochondrial oxidative stress has shown beneficial effects. Increased expression of mitochondrial superoxide dismutase (SOD) has been reported to improve synaptic function and cognitive performance in AD models (Dumont et al., 2009). Similarly, mitochondrial-targeted antioxidants such as mitoQ (Mitoquinone), SS peptides, and SOD mimetics have shown protective effects against Aβ-induced synaptic damage (Ma et al., 2011). At the same time, it is important to note that ROS also play physiological roles in synaptic signaling, and so reducing ROS excessively could interfere with normal synaptic functionality (Lin and Beal, 2006). Aβ further contributes to mitochondrial dysfunction by impairing the ETC, thereby reducing ATP production and increasing ROS generation (Brookes et al., 2004; Crouch et al., 2008; Kaur et al., 2015). Most of this mechanistic evidence derives from *in vitro* studies and transgenic rodent models. Although some supporting evidence has been observed in human AD tissue, such data are still relatively limited.

In addition to ROS, mitochondrial Ca^2 +^ handling plays an important role in synaptic regulation. Mitochondria act as Ca^2 +^ buffers in presynaptic and dendritic compartments, supporting both ATP production and neurotransmission (Zorzano and Claret, 2015). Regulated Ca^2 +^ uptake helps maintain mitochondrial potential and activity, but excessive accumulation can lead to increased ROS production and cellular damage, often associated with excitotoxic conditions (Nicholls, 2009). Disrupted Ca^2 +^ homeostasis also affects synaptic plasticity, as both ROS levels and Ca^2 +^ signaling needs to be tightly regulated for processes such as long-term potentiation (LTP) (Lee et al., 2010). When mitochondrial buffering capacity is exceeded, this triggers mitochondrial membrane permeabilization and cell death (Brawek et al., 2014).

Mitochondrial defects occur prior to Aβ plaque accumulation in the brains of AD mouse models. Swerdlow et al. (2014) first proposed the mitochondrial cascade hypothesis, positing that mitochondrial dysfunction is an early driver in AD pathogenesis. Consistent with this concept, impaired mitochondrial function not only shifts amyloid precursor protein (APP) processing towards greater Aβ production but also promotes tau hyperphosphorylation and heightens cellular vulnerability to Aβ and tau-mediated toxicity, ultimately contributing to neurodegeneration (Zhao and Zhao, 2013; Swerdlow et al., 2014). Oxidative stress represents a central pathway through which mitochondrial dysfunction exerts its effects. In AD, elevated oxidative damage to proteins and lipids disrupts their normal function and progressively impairs neuronal viability. Moreover, oxidative stress has been shown to upregulate key Aβ-producing enzymes, including BACE1 (the brain’s β-secretase) and PS1 (a central component of γ-secretase) (Tamagno et al., 2002). Oxidative stress is also a contributing factor to both Aβ- and tau-mediated neurotoxicity. Although motile mitochondria perform these essential functions, stationary docked mitochondria are also required to meet the high energy demand of axonal and synaptic functions. Docked mitochondria act as fixed energy sources that provide a steady ATP supply required for Na^+^/K^+^-ATPase function, rapid action potential conduction, and efficient synaptic transmission (Sheng, 2014). Synaptic membrane proteins, long recognized for inhibiting SNARE-mediated vesicle docking and neurotransmitter release, have also been implicated in regulating mitochondrial anchoring. Syntaphilin is one such protein critical to this process: acting as a specialized mitochondrial tether, it anchors these organelles within the axonal domain (Kang et al., 2008). Following retrograde transport of damaged mitochondrial tether towards the soma, syntaphilin detachment triggers PARK2/Parkin-dependent mitophagy. Notably, this mechanism, dependent on syntaphilin, is highly activated in early dysfunction of cortical neurons observed in AD (Lin et al., 2017).

Mitochondrial dysfunction is becoming a well-recognized commonality in both familial and sporadic AD (Swerdlow et al., 2014). Consistent with this observation, alterations in mitochondrial mass, enzyme activity, and mtDNA were observed in the brains of AD patients, which are associated with morphological changes, such as cristae disruption, accumulation of osmiophilic material inside mitochondria, and alterations in the size of the organelles (Silva et al., 2012). Glucose uptake by neurons is impaired in the brains of AD patients, potentially reflecting impaired enzyme efficiency within mitochondrial OXPHOS as well as the TCA cycle (Kapogiannis and Mattson, 2011). Elevated oxidative stress has been detected in the brains of AD patients even in the early stages of the disease (Nunomura et al., 2001). This degree of oxidative stress correlates with cognitive deficits and symptomatic progression from mild cognitive impairment (MCI) to AD, highlighting a strong association between mitochondrial dysfunction and the development of AD (Ansari and Scheff, 2010; Verma et al., 2023).

**FIGURE 1:**
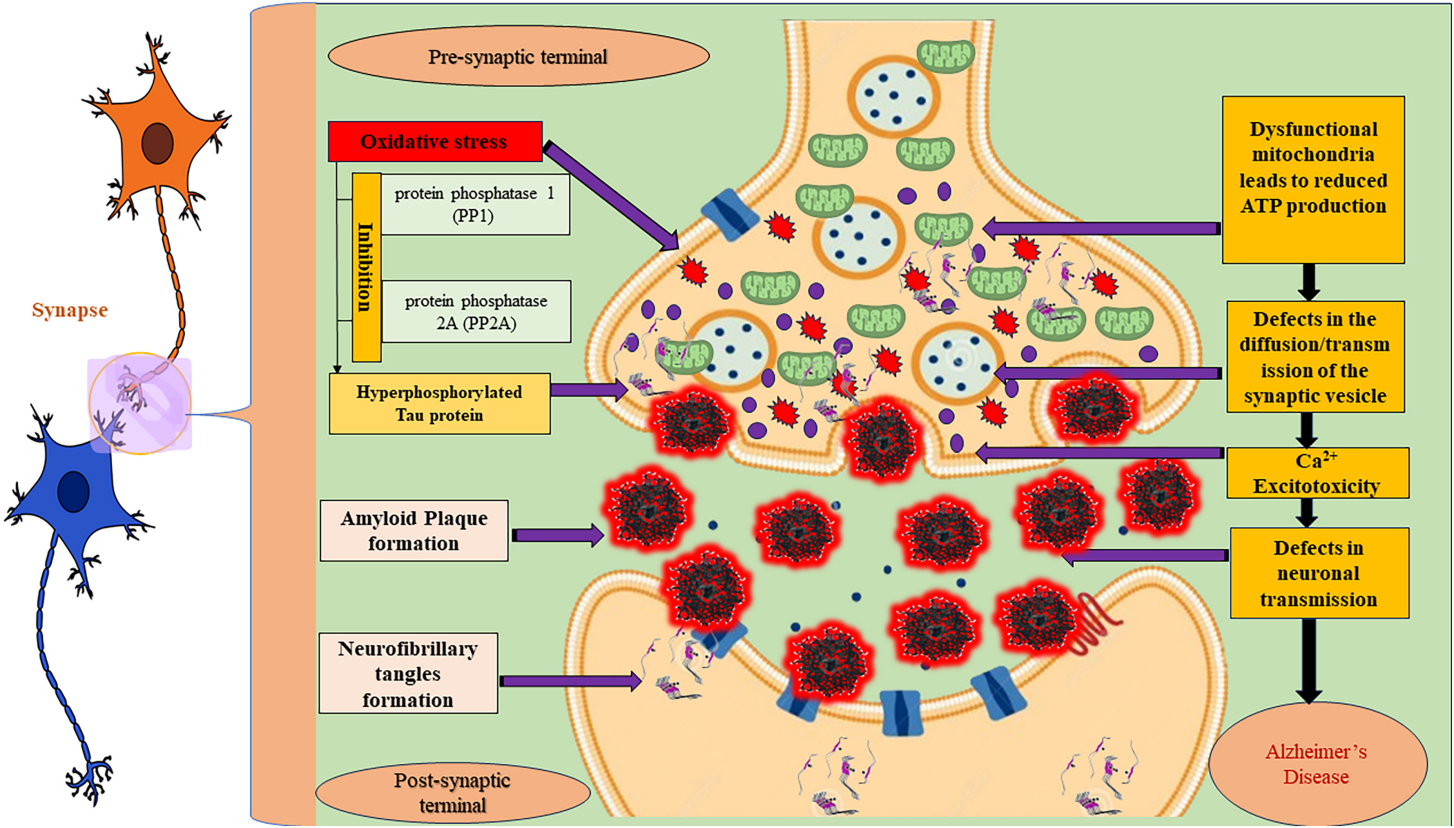
Excessive accumulation of Aβ and hyperphosphorylated tau disrupts mitochondrial function and integrity. Impaired mitochondrial Ca^2 +^ homeostasis leads to increased ROS production, thereby exacerbating oxidative stress and promoting excitotoxicity and neuronal vulnerability. Together, these changes compromise synaptic structure and function, ultimately contributing to the progressive synaptic degeneration and cognitive decline in AD.

Thus, mitochondrial dysfunction emerges as a key driver of synaptic failure, neurodegeneration, and the transition toward AD. Initial dysfunction in OXPHOS, ROS modulation, Ca^2 +^ homeostasis, mitochondrial fission-fusion dynamics, and mitophagy compromise neuronal energy supply and increase susceptibility to Aβ and tau toxicity. Such events increase oxidative stress, hinder synaptic plasticity, and precipitate cognitive decline. Since mitochondrial integrity has a strong correlation with synaptic health, therapeutic strategies capable of restoring mitochondrial bioenergetics, modulating ROS levels and Ca^2 +^ buffering, or promoting efficient mitochondrial turnover via mitophagy are highly promising in ameliorating disease progression and improving clinical outcomes (Yusuf et al., 2025; Mary et al., 2023). Along with mitochondrial bioenergetics, the stability of synapses also depends on cytoskeletal remodeling, which supports spine structures, intracellular transport, and activity-dependent synaptic repair (Figure 1). However, it is important to note that the majority of therapeutic evidences in this area remains confined to preclinical studies, and the clinical translation of mitochondrial-targeted strategies in AD has yet to yield consistent outcomes. Furthermore, a critical and unresolved debate in the field concerns whether mitochondrial dysfunction acts as an early driver of pathology or arises as a consequence of Aβ and tau accumulation. The mitochondrial cascade hypothesis (Swerdlow et al., 2014) proposes that inherent mitochondrial deficits initiate disease progression, whereas the amyloid cascade hypothesis considers mitochondrial impairment secondary to Aβ toxicity. This distinction has important therapeutic implications; an early targeting of mitochondrial function may be beneficial only if it represents a primary event, if not, such approaches may have limited impact. Current evidences suggest that both processes likely interact in a stage-dependent, feed-forward manner.

## 3 Cytoskeletal remodeling in synaptic function and dysfunction in AD

Synapses in the nervous system depend on cytoskeletal components, particularly microtubules and actin filaments, for their structural integrity and functional organization. These dynamic structural polymers not only coordinate the activities required for synaptic growth, signaling, and repair, while also providing structural support to axonal terminals and dendritic spines. Actin filaments control the shape, mobility, and stability of dendritic spines by constant cycles of polymerization and depolymerization; they make up around 90% of the cytoskeletal mass of dendritic spines (Hotulainen and Hoogenraad, 2010). Synaptic functionality is strengthened during LTP by fast actin polymerization, which increases spine head size, improves postsynaptic density (PSD) integrity, and enables AMPA receptor anchoring. Microtubules are primarily localized to dendritic shafts, but during activity-dependent remodeling, these filaments transiently enter dendritic spines, where they carry messenger RNAs, PSD95, and mitochondria required for synaptic repair and spine development (Hu et al., 2008; Miryala et al., 2022). Remarkably, actin filaments and microtubules are not autonomous cytoskeletal systems; rather, synaptic integrity depends on their coordinated regulation. An early molecular driver of synaptic failure in AD is cytoskeletal dysregulation, which is disrupted prior to overt neuronal loss (Dent et al., 2011; Pelucchi et al., 2020).

In addition to the structural polymers, motor proteins play a key regulatory role in cytoskeletal function by generating force and facilitating intracellular transport. Myosin motors interact with actin filaments, where kinesin and dynein use microtubules as motor tracks. While processive myosin like myosin V and VI facilitate the short-range transport of synaptic vesicles, receptors, and endocytic cargos within dendritic spines, myosin II controls actin contractility and spine tension, impacting spine shape, motility, and synaptic stability (Hirokawa et al., 2010). In AD, the dysregulated RhoA/ROCK signaling pathway promotes spine retraction and synaptic loss through increasing myosin II-dependent actin contractility. Studies have demonstrated the pharmacological inhibition of this pathway partially restores synaptic structures (Henderson, 2019; Glotfelty et al., 2023). Conversely, kinesin and dynein motor proteins facilitate the long-range transportation of mitochondria, synaptic proteins, messenger RNAs, and signaling complexes along microtubules, thereby ensuring synapse development, maintenance, and activity-dependent structural remodeling. Research has shown that abnormal tau proteins hinder the movement of kinesin-1 by adhering to microtubules and blocking motor progression. This, in turn, halts the transport of cargo to dendrites and axons, which occurs prior to synaptic impairment in AD models (Morris et al., 2011; Durairajan et al., 2024). Furthermore, hyperphosphorylated tau undergoes conformational changes that promote its self-aggregation into paired helical filaments (PHFs) and ultimately neurofibrillary tangles (NFTs). Soluble tau oligomers (tauO), which are the pre-tangle species are now recognized as the primary synaptoxic form, selectively targeting presynaptic GABAergic terminals and interacting with Fyn kinase to facilitate rapid internalization at synaptic sites, where they degrade the local cytoskeleton and destabilize dendritic spines (Kadamangudi et al., 2024; Verma et al., 2024b). According to Pelucchi et al. (2020) and Miryala et al. (2022), the precise spatial control of synaptic assembly and repair is ensured by coordinated actin-microtubule interactions. Furthermore, the research demonstrates that tauO show preferential selectivity to human presynaptic compartments demonstrating a significant preference for GABAergic (inhibitory) synapses over excitatory ones. Electrophysiological evaluations indicate that acute tauO exposure specifically enhances inhibitory currents, whereas proteomic mapping demonstrates a high-affinity interaction between tauO and presynaptic vesicle proteins, suggesting that early synaptic failure in tauopathies is caused by a targeted disruption of the presynaptic inhibitory machinery rather than a widespread postsynaptic loss (Kadamangudi et al., 2026).

Moreover, actin-binding proteins like cofilin, Arp2/3, profilin, drebrin, and cortactin control actin dynamics in dendritic spines, enabling rapid changes in spine morphology. Notably, there is mounting evidence that early neurodegenerative disease is associated with cofilin dysregulation, which in turn promotes the production of cofilin-actin rods that hinder intracellular trafficking and synaptic integrity (Bamburg and Bernstein, 2016; Shehjar et al., 2024). Microtubule stability and targeting to active synapses are controlled by microtubule-associated proteins (MAPs), including tau, MAP2, and EB3. Some linker proteins, such as spectraplakins, IQGAP1, and CLIP-170, mediate the functional interaction between microtubular networks and actin filaments, facilitating synaptic plasticity and homeostatic restoration (Dent et al., 2011; Swiech et al., 2011). Importantly, excitatory and inhibitory synapses differ markedly in their cytoskeletal organization. The stability and plasticity of excitatory glutamatergic synapses are greatly influenced by actin turnover, which is abundant in dendritic spines at these synapses. In inhibitory GABAergic synapses, on the other hand, scaffolding associated with the shaft and microtubule-based transport are more important. Synapse loss correlates more strongly with cognitive decline than cell death, according to consistent neuropathological investigations that show that AD primarily affects excitatory synapses in the hippocampus and cortex (Terry et al., 1991; Selkoe, 2002).

The molecular-level connection between synaptic activity and cytoskeletal remodeling is established by activity-dependent Ca^2+^ influx through NMDA receptors. During LTP, controlled phosphorylation of cofilin is brought about by an increase in intracellular Ca^2 +^, which in turn stimulates CaMKII and downstream cofilin kinase (LIM kinase) signaling. At the same time that RhoA-mediated contractile signaling is suppressed, activation of Rac1 and Cdc42 (two Rho family GTPases) stimulates actin branching and spine growth. According to the research conducted by Bosch and Hayashi (2012), Murakoshi et al. (2011), and Zhang et al. (2021), certain pathways transform short-lived synaptic activity into long-term structural changes that support synapse strengthening and memory formation. In addition to this, cytoskeletal remodeling is critical for synaptic regeneration after metabolic, oxidative, or excitotoxicity load. Restoration of synaptic structures and functions is facilitated by actin-driven spine reformation in conjunction with microtubule-dependent transport of repair-associated cargos (Murakoshi et al., 2011; Bosch and Hayashi, 2012; Zhang et al., 2021). Another mechanism supporting long-term synaptic regeneration is local protein synthesis within the dendrites. This process relies on intact microtubule-based transport of messenger RNAs and ribonucleoprotein complexes (Bramham and Wells, 2007; Kapitein and Hoogenraad, 2015). By stimulating cytoskeletal pathways, neurotrophic signaling amplifies the healing processes that encourage spine stability and regrowth. According to the study outcomes by Shehjar et al. (2024), Aβ oligomers impede intracellular trafficking by interfering with actin-binding proteins, resulting in actin depolymerization, hyperactivation of cofilin, and the production of cofilin-actin rods. Cognitive impairment in AD patients is strongly associated with a dramatic decrease in drebrin, a critical F-actin stabilizer that is abundant at the excitatory synapses, in the hippocampus. At the same time, tau hyperphosphorylation disrupts synaptic maintenance and repair by dissociating tau from microtubules, which destabilize microtubules and impairs axonal transport (Morris et al., 2011). Furthermore, impairment in microtubule invasion into the dendritic spines is another early pathogenic characteristic of AD. The structural flexibility and activity-dependent strengthening of the spine are supported by dynamic microtubule invasion in the healthy synapse. These invasions have been found to be diminished in AD models because of aberrant tau build-up and tubulin instability caused by Aβ (Morris et al., 2011; Fernandez-Valenzuela et al., 2020). Moreover, according to Kadamangudi et al. (2024), Aβ oligomers activate Fyn kinase in synapto-neurosomes isolated from humans, priming the synapse for rapid binding and internalization of tau oligomers; this toxic interaction subsequently degrades the local cytoskeleton and leads to dendritic spines loss. Since activity-dependent spine reinforcement relies on microtubule-based cargo transport, disruption of microtubule-based cargo transport therefore directly contributes to synaptic failure and represent a critical factor in early synaptic vulnerability in AD as reviewed previously (Verma et al., 2024b).

**FIGURE 2:**
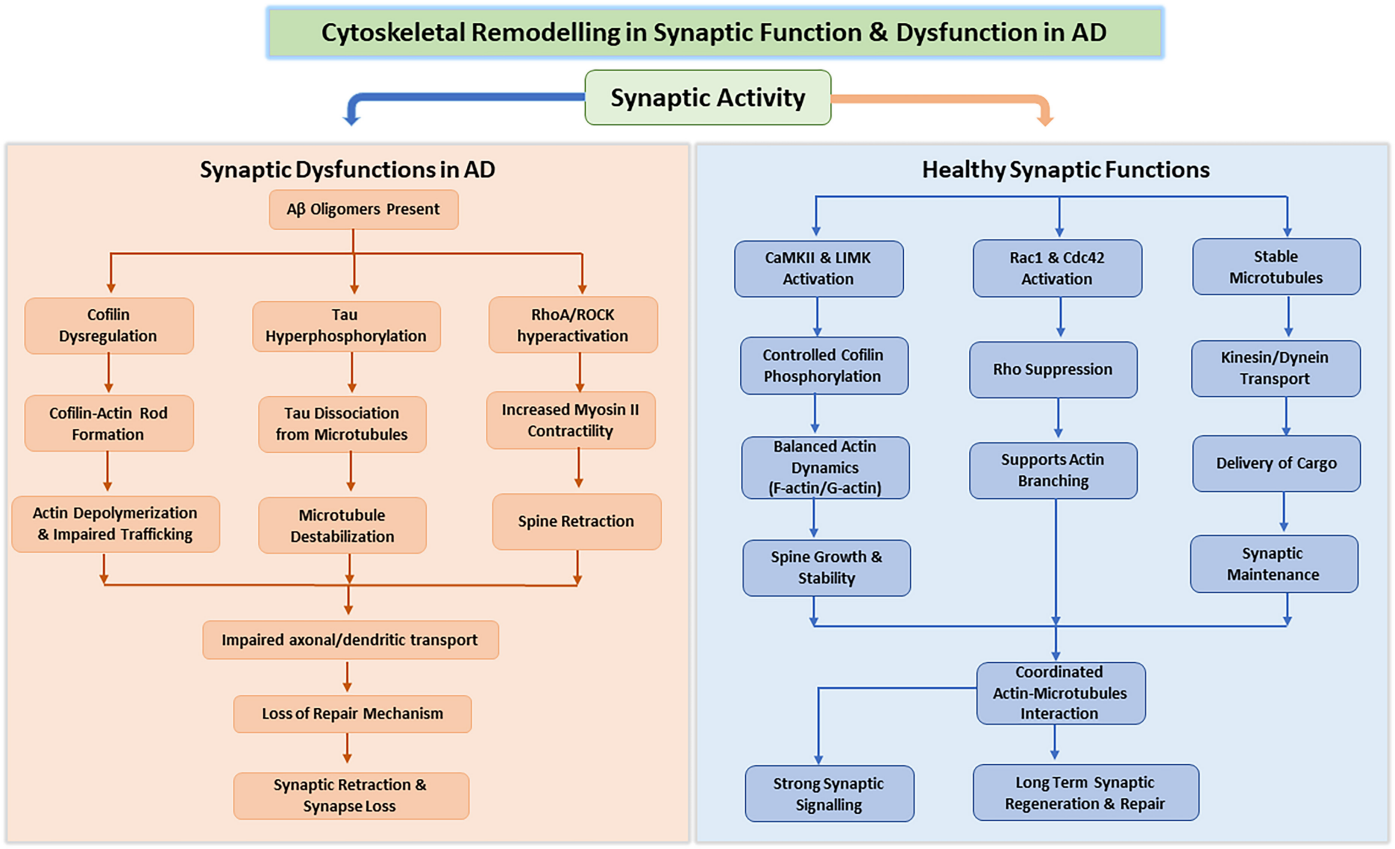
In healthy neurons, CaMKII–LIMK and Rac1/Cdc42 signaling maintain actin dynamics and microtubule stability, supporting efficient synaptic transmission. In AD, Aβ-induced cofilin dysregulation, tau pathology, and RhoA/ROCK activation disrupt cytoskeletal integrity. These changes impair intracellular transport and lead to dendritic spine loss and synaptic degeneration.

Alongside neurons, cytoskeletal dysfunction in glial cells also plays a significant role in the synaptic pathology observed in AD. Microglia promote excessive and abnormal synaptic pruning through dysregulated actin remodeling, while astrocytes impair synaptic shielding and metabolic support through altered actin dynamics. It has been noted that Aβ triggers microglial RhoA/ROCK signaling, which exacerbates actin contractility and the abnormal removal of functional synapses (Hong et al., 2016). The importance of maintaining healthy cytoskeleton at synapse has generated growing interest in therapeutic strategies that aim to improve actin dynamics, motor protein function, and microtubule stability (Figure 2). According to Pelucchi et al. (2020) and Pa et al. (2024), restoring spine structures and synaptic plasticity in AD models can be achieved by stabilizing F-actin, regulating cofilin phosphorylation, and activating Rac1/Cdc42 signaling (Pelucchi et al., 2020; Pa et al., 2024). Numerous preclinical studies have shown that microtubule-stabilizing drugs, such as epothilone-based and next-generation brain-penetrant compounds, can ameliorate tau pathology and enhance axonal transport (Brunden et al., 2010; Fernandez-Valenzuela et al., 2020; Zhao et al., 2023). Further, physiological plasticity can be impaired by over-stabilizing cytoskeletal elements or motors, highlighting the importance of precise, context-dependent regulation.

Taken together, the evidence collectively underscores that synaptic stability in AD is maintained through the coordinated dynamics of actin filaments and microtubules, regulated by motor proteins, with early disruption of this coupling contributing to synaptic susceptibility. Therapeutic strategies targeting cytoskeletal-motor connections such as F-actin stabilization, cofilin regulation, and microtubule-stabilizing agents show potential in preserving synaptic integrity and mitigating cognitive decline, though clinical validation remains an essential next step. It should be noted that most cytoskeletal evidence derive from transgenic mouse model and post-mortem tissue, direct interventional data in human AD patients remain limited. In addition to intracellular mechanisms such as cytoskeletal remodeling, synaptic function is also influenced by intracellular communication mediated by extracellular vesicles, particularly exosomes.

## 4 Exosomes in synaptic communication and dysfunction in AD

Intercellular communication in mammals occur either through direct contact between cells via surface proteins or at a distance via secreted molecules (such as exosomes) binding to receptors. Exosomes serve as natural messengers and emerge as a means of transferring their cargo (Chivet et al., 2012; Kaur et al., 2021; Cleary et al., 2025). Exosomes are 30–200 nm sized small extracellular vesicles derived from endosomes and carry proteins, lipids, nucleic acids, and various signaling molecules as cargo. These are released by almost all types of cells in the extracellular space, facilitating communication between distant cells (Kalluri and LeBleu, 2020). Likewise, neuronal exosomes are important mediators of intercellular communication between neurons and between neurons and glial cells, and are potential modulators of gene expression underlying both pathological and physiological conditions in the brain. They are also being explored as diagnostic biomarkers and as delivery platforms for therapeutic agents, given their capacity to cross the BBB (Zhang and Yang, 2018; Delpech et al., 2019). Interestingly, the dual role of exosomes released from the damaged neurons has been reported in the case of AD. They can either exacerbate degeneration by spreading the β/γ-secretase, Aβ peptide, tau, and APP to healthy neurons, resulting in the accumulation of Aβ and accelerating toxicity at the synapses, or they can aid in Aβ clearance by delivering it to microglial lysosomes for degradation, thereby supporting synaptic plasticity as reviewed previously (Kaur et al., 2021). Given their ability to transfer bioactive cargo between cells, exosomes occupy a pivotal position at the intersection of synaptic communication, neuroinflammation, and AD pathogenesis.

Neuronal exosomes carry neurotransmitter transporters, which are delivered to presynaptic active zones to regulate local neurotransmitter availability, while postsynaptic exosomes transport AMPA receptors and NMDA receptors to dendritic spines in an activity-dependent manner to modulate synaptic strength (Brown et al., 2007). In addition, neuronal and glial exosomes, enriched with regulatory RNAs, including microRNAs such as miR-124 (especially miR-124a/miR-124-3p), act as a post-transcriptional regulator of synaptic function by targeting genes involved in synaptic plasticity, neurotransmitter release, and neural differentiation (Xu et al., 2022). On the other hand, neuronal exosomal miR-132 maintains the integrity of the BBB by suppressing eukaryotic elongation factor 2 kinase (eef2k) and increasing vascular endothelial-cadherin, which stabilizes the synaptic microenvironment by ensuring proper delivery of oxygen and glucose and preventing toxic leakage, thereby supporting efficient synaptic plasticity and transmission (Xu et al., 2017). Furthermore, neuronal-derived exosomes are involved in various functions of the brain, including synaptic plasticity, neurogenesis, inflammation, pruning of excess synapses, synaptogenesis, and axon guidance (Budnik et al., 2016; Zappulli et al., 2016). Exosomal miRNAs also affect synaptic function by regulating local protein synthesis in dendrites and synaptic regions. miR-134 and miR-138, for example, are closely linked to dendritic spine structure and size, and in turn influence synaptic strength. When their expression levels are dysregulated, spine maturation and stability can be compromised. These miRNAs also shape synaptic plasticity by influencing actin dynamics and receptor internalization at the synapse as reviewed previously (Kaur et al., 2024).

In contrast, brain-derived exosomes have been reported to spread pathological proteins such as APP, Aβ, tau, and α-synuclein, which are involved in neurodegenerative conditions (Torres and Lee, 2023). Similarly, supporting brain cells, such as astrocytes and microglia, are involved in the spread of Aβ and tau pathology by releasing exosomes that facilitate the intercellular transmission of these toxic proteins (Ruan, 2022). Astrocyte-derived exosomes have been shown to contain elevated levels of oligomeric Aβ and tau in an animal model of AD, whereas exposure of astrocytes to Aβ induced increased expression of tau and phosphorylated tau, promoting the release of exosomes that carry these pathogenic proteins and thereby facilitating the propagation of neurodegenerative pathology throughout the brain (Rosas-Hernandez et al., 2019). Studies have shown that exosomes from AD brains carry oligomeric Aβ and enter recipient neurons through dynamin-dependent endocytosis, delivering Aβ directly into the endosomal-lysosomal system (Sun and Chen, 2024). Inside neurons, these exosomes stabilize Aβ, promote its intracellular accumulation, and trigger downstream toxicity by disrupting synaptic signaling and membrane integrity. However, silencing key ESCRT proteins (TSG101, VPS4A) reduces exosome formation, thereby blocking intercellular Aβ trafficking. Inhibiting exosome uptake similarly prevents the prion-like spread of Aβ and protects neurons. Collectively, these findings establish, exosome-mediated intracellular delivery and propagation of Aβ represent a central pathway driving AD progression (Sinha et al., 2018). Exosomal Aβ(1-42) affects key receptors and plasticity pathways, significantly hindering connectivity at the synapse. Upon release, it binds to NMDA receptors, especially GluN2B subunits, leading to its aberrant NMDA receptor activation, increased Ca^2 +^ influx, excitotoxicity, and oxidative stress. At the same time, it enhances the internalization of AMPA receptors (GluA1/GluA2), which reduces excitatory synaptic transmission. It also disrupts Ca^2 +^-dependent signaling via kinases such as CaMKII and PKA, and causes mitochondrial dysfunction (Zhang et al., 2022). These receptor-level changes diminish LTP by degrading vital postsynaptic density proteins. Likewise, exosomal Aβ(1-42) exacerbates long-term depression (LTD) by elevating calcineurin (PP2B) activity, accelerating AMPA receptor endocytosis, and reducing synaptic stability. Collectively, these effects result in loss of plasticity, overall synaptic weakening, and ultimately the memory impairment and cognitive decline that are hallmarks of AD (Tu et al., 2014; O’Riordan et al., 2018). These mechanistic findings are primarily derived from *in vitro* electrophysiological and biochemical studies, with supporting evidence from rodent models. Importantly, limitations should be considered when interpreting this evidence. First, the protective versus pathological role of exosomes in AD remains context-dependent and unresolved. While some studies suggest that neuronal exosomes promote Aβ clearance via microglial uptake, others indicate that they facilitate the spread of Aβ and tau, depending on disease stage, cellular origin, and cargo composition. This duality limits broad generalization across studies. Second, exosome isolation methodologies vary considerably across studies. Ultracentrifugation, precipitation-based kits, and size-exclusion chromatography yield vesicle populations of differing purity and cargo composition, making cross-study comparisons unreliable. Standardization of isolation and characterization protocols is essential before exosomal findings can be translated into reliable diagnostic or therapeutic applications.

Likewise, oligomeric Aβ, tau oligomers and aggregates are reported to spread via exosomes through synaptic connections between the neurons. Study of Kadamangudi and their group showed that Aβ oligomers can promote the binding and internalization of tau oligomers at synapses, facilitating their spread between neurons (Kadamangudi et al., 2024). These pathological tau oligomers are released at the presynaptic terminals through unconventional secretory pathways, independent of the ER/Golgi, and are internalized by postsynaptic neurons through endocytosis at the synaptic sites, where they disrupt synaptic homeostasis, leading to microtubule destabilization and mislocalization across axonal and synaptic compartments. Furthermore, in dendritic spines, these tau oligomers orchestrate the prion-like aggregation of endogenous tau, thereby accelerating synaptic tau aggregation (Wang et al., 2017; Wei et al., 2022). This causes impairment of synaptic vesicle trafficking, reduced receptor stability (NMDA/AMPA), and loss of synaptic plasticity. Internalized exosomes can be re-released at the downstream synapses, enabling step-wise trans-synaptic propagation of tau pathology. Thus, exosome-mediated tau transfers directly couples tau spread with progressive synaptic dysfunction and network-level disconnection in AD. Apart from local neuronal communication, circulating (serum-derived) exosomes have been shown to reflect changes in pathways associated with synaptic functions. In AD, proteins linked to energy production and Aβ clearance are significantly reduced, while cytoskeletal proteins become dysregulated (Verma et al., 2025).

Beyond neuronal communication, exosomes also mediate bidirectional communication between neurons, astrocytes, and microglia, thereby influencing neuroinflammatory reactions and synaptic functions. Under stressful conditions, exosomes derived from astrocytes regulate synaptic activity by maintaining extracellular glutamate homeostasis and delivering neuroprotective cargo molecules, such as apolipoprotein D, which promote neuronal survival (Vasile et al., 2017; Pascua-Maestro et al., 2019). Microglial exosomes, on the other hand, modulate immune signaling and neurotransmission by delivering inflammatory miRNAs and bioactive lipids such as sphingosine and ceramides. Under physiological conditions, glial cell-derived exosomes enhance synaptic repair and pruning. However, during chronic neuroinflammation, they can shift towards exacerbating synaptic injury via innate immune receptors, like TLR4 and NLRP3 (Antonucci et al., 2012; Pascual et al., 2020). Recent studies have shown that neuronal-derived exosomes also aid in the conformational change of extracellular Aβ, converting it from a nontoxic to a toxic fibrillar form. However, via phosphatidylserine-dependent pathways, microglia efficiently recognize and internalize these Aβ-exosome complexes, followed by the trafficking of Aβ to lysosomes, where it undergoes lysosomal degradation, thereby reducing the extracellular Aβ burden (Yuyama et al., 2012).

Thus, in AD, exosomes can either protect or impair synaptic functions. Toxic exosomes disseminate Aβ, tau, and inflammatory signals, which impede synaptic transmission and promote degeneration. Protective exosomes, on the other hand, facilitate the removal of Aβ peptides and deliver neurotrophic and synapse-supporting molecules that support synaptic recovery. This dual nature makes exosomes important regulators of synaptic fate and promising targets for AD therapeutics (Kaur et al., 2021; Verma et al., 2024a,^2025^). Besides exosomes-mediated signaling, synaptic dysfunction in AD is also influenced by neuroinflammatory responses involving peripheral immune cells such as neutrophils.

## 5 NETosis-driven neuroinflammation and synaptic dysfunction in AD

(i) NETosis and its role in neuroinflammation:

NETosis is one of the innate defense mechanisms executed by neutrophils, in which they release their decondensed DNA that traps and kills invading pathogens through released cytotoxic proteins. Histones, myeloperoxidase, neutrophil elastase, cathepsin G, proteinase 3, and others are secreted cytotoxic NET components that, in chronic conditions, lead to inflammation (Delgado-Rizo et al., 2017; Pietronigro et al., 2017). Under normal conditions, NETosis serves a host-protective function, but excessive NETosis leads to tissue damage, chronic inflammation, and related complications. Beyond pathogen-driven activation, NETosis can also be triggered by damage-associated molecular patterns (DAMPs), and other biological stimuli, potentially leading to a chronic inflammatory condition. NETosis releases cytotoxic nuclear and granular constituents, including proteins, to kill the pathogen extracellularly (Almyroudis et al., 2013) and causes acute inflammation. However, in the brain, these constituents, if they lead to a chronic state, can also damage surrounding neural tissue and contribute to neurodegeneration.

Neuroinflammation is the immune response of the CNS, mediated by activated immune cells and endothelial cells via the secretion of various inflammatory molecules and cytokines (DiSabato et al., 2016). Neutrophils are among the peripheral immune cells that cannot penetrate the brain due to the presence of the BBB, which regulates the flow of leukocytes and vital nutrients into the CNS and facilitates the removal of neurotoxic substances from the brain into the blood (Blair et al., 2015; Pietronigro et al., 2017). In response to AD pathogenesis, microglia and astrocytes become overactivated and release pro-inflammatory cytokines, resulting in increased expression of ICAM-1, to which peripheral immune cells attach and migrate to the CNS via a disrupted BBB (Greenwood et al., 2002). Under physiological conditions, neutrophils are not found in the brain region, but in pathological conditions, they migrate into the brain parenchyma via breached BBB, driven by chemotactic signals and inflammatory mediators. In the AD mouse model, neutrophils are found to actively migrate toward Aβ plaques, exhibiting increased vascular adhesion and motility, and become activated, contributing to neurovascular inflammation and disease progression (Baik et al., 2014). In another study, neutrophils were found to be involved in AD pathophysiology via binding to high-affinity LFA-1 and crossing the BBB, leading to neurodegeneration (Zenaro et al., 2015). These studies collectively suggest that NETosis may contribute to synaptic degeneration as part of AD pathology. NETosis is critically regulated by peptidyl-arginine deiminase 4 (PAD4), which citrullinates histones to facilitate chromatin decondensation and NET release. PAD4 inhibition has been shown to reduce NET formation and represents a potential therapeutic target in neuroinflammatory conditions. Synaptic loss is a hallmark of AD, and NETs may therefore actively contribute to this degenerative process. In the presence of NETs, macrophages have been found to secrete pro-inflammatory cytokines such as TNF-α and IL-8, and via the activated NLRP3 inflammasome, they secrete IL-1 and IL-18 (Dömer et al., 2021). NET formation in the brain parenchyma and cerebral vessels may damage cerebral vessels and surrounding neurons, thereby exacerbating neuroinflammation (Dong et al., 2018). Neutrophils have been found to damage the neurons through the release of proteases and extracellular DNA (Allen et al., 2012).

(ii) Crosstalk between NETosis and synaptic damage:

Numerous studies have found that NETs are not merely disease markers, but also lead to neurodegeneration by causing inflammation. The direct role of NETosis in synaptic loss remains unknown; however, elevated NETosis in AD may lead to neurodegeneration through synaptic loss, facilitated by the release of NET-derived histones and proteases as reviewed recently (Rao R. et al., 2026). Extracellular histones can act as DAMPs and become toxic (Richards et al., 2023). It can also activate microglia, resulting in the release of pro-inflammatory cytokines and aberrant phagocytosis at the synapse. Moreover, NET-derived enzymes such as MPO and proteases like NE can cause severe inflammation by degrading the ECM (Zhu et al., 2021). NETs release metalloproteinases (MMPs), overexpression of which can degrade the ECM surrounding the synapse and lead to structural instability of the synapse. Under physiological conditions, well-regulated MMPs contribute to synaptic plasticity, but their over activation can disrupt the synaptic integrity and neuronal circuitry (Huntley, 2012). Other NET components, including NE, degrade ECM proteins such as proteoglycans, collagen, and elastin, activating MMPs and deactivating Tissue Inhibitors of Metalloproteinases (TIMPs) (Itoh and Nagase, 1995). In this way, NETosis may contribute to synaptic damage, as it has been shown to be a significant contributor to AD progression, but its mechanism of action in this context remains underexplored. Thus, these findings advocate the need for therapeutic approaches that can counteract inflammation-driven synaptic damage and restore synaptic homeostasis and attenuate neuroinflammation in AD.

Although direct *in vivo* evidence linking NETosis specifically to synaptic terminal loss in AD is still lacking, emerging evidence increasingly support a well-defined mechanistic framework. Components of NETs, particularly histones, can activate microglial TLR/NF-κB signaling, which in turn promotes complement-driven synaptic pruning. At the same time, NET-associated proteases degrade the surrounding extracellular matrix, weakening synaptic structure (Zenaro et al., 2015; Wen et al., 2024). In addition, NET-induced disruption of the BBB allows greater infiltration of peripheral immune cells, further sustaining neuroinflammation and accelerating synaptic damage.

## 6 Phytochemicals as multi-targeted neuroprotective agents in AD

In recent years, there has been growing interest in natural compounds as complementary and alternative approaches for the treatment of neurological disorders. Among these, phytochemicals have gained immense attention as potential neuroprotective agents for the treatment of various neurological disorders due to their ability to target multiple molecular mechanisms simultaneously (Table 1; Youdim and Joseph, 2001; Kim et al., 2010; Kaur et al., 2017; Alum et al., 2025). Since phytochemicals have antioxidative, anti-inflammatory, and neurogenic properties, they have the potential to counteract the effects of oxidative stress, neuroinflammation, and apoptosis (Kaur et al., 2018; Jawale and Jain, 2024). These substances have been reported to modulate neuronal functions by interacting with a range of cellular processes. Although not all phytochemicals have the ability to traverse the BBB, certain phytochemicals do demonstrate BBB permeability (Venkatesan et al., 2015; Sánchez-Martínez et al., 2022; Singh et al., 2025; Yuan et al., 2025). These properties suggest their potential relevance in conditions such as ischemic stroke (Xu et al., 2021), age-related cognitive decline (Sharifi-Rad et al., 2022; Alum et al., 2025), PD (Balakrishnan et al., 2021), and traumatic brain injury (TBI) (Kaur et al., 2017; Hasan et al., 2024) though clinical evidence remains variable across these conditions. Consequently, phytochemicals may have therapeutic potential in the treatment of complex diseases such as AD, distinct from drugs that are synthesized and typically act through a single mechanism. The following sub-sections explore the mechanisms and applications of phytochemicals toward neuroprotection including:

**TABLE 1:** Phytochemicals with their source and main primary mechanisms.

Sr. No.	Name	Source	Primary mechanism	Reference(s)
1.	Curcumin	Turmeric (*Curcuma longa*)	Inhibits amyloid aggregation; Nrf-2 activation	Lim et al., 2001; Reddy et al., 2018
2.	Resveratrol	Grapes, berries (*Vitis vinifera*)	SIRT1/Nrf-2 activation; anti-oxidative stress	Farhan and Rizvi, 2023; Shen et al., 2016
3.	Quercetin	Onions, apples (*Allium cepa*, *Malus domestica*)	Free radical scavenging; anti-inflammatory	Dehghani et al., 2021; Mirza et al., 2023
4.	EGCG	Green tea (*Camellia*)	Anti-amyloidogenic; PKC/survival pathways	Fernandes et al., 2021; Rezai-Zadeh et al., 2005
5.	Bacosides A/B	*Bacopa monnieri*	Enhances neuronal kinase; synaptic repair	Paul et al., 2025; Valotto Neto et al., 2024
6.	Ginsenosides	Ginseng (*Panax ginseng*)	BDNF-TrkB signaling; anti-apoptotic	Zarneshan et al., 2022; Zheng et al., 2018
7.	Berberine	Goldenseal, Barberry (*Berberis vulgaris*)	Nrf-2/HO-1 pathway; neuroinflammation inhibition	Fan et al., 2019; Hsu et al., 2012
8.	Asiaticoside	Gotu kola (*Centella asiatica*)	Oxidative stress reduction; cognitive support	Wong et al., 2021
9.	Ginkgolides	*Ginkgo biloba*	Improves microcirculation; PAF antagonism	Yao et al., 2026
10.	Sulforaphane	Broccoli, cruciferous vegetables (*Brassica oleracea*)	Nrf-2 induction; detox enzyme upregulation	Kumar and Khanum, 2012; Zhao et al., 2006

(i) Mechanistic basis of neuroprotection by phytochemicals

Antioxidant defense: as discussed in Section “2 Mitochondrial bioenergetics and synaptic dysfunction in AD,” oxidative stress is a central driver of synaptic dysfunction in AD; phytochemicals counteract this through multiple complementary antioxidant mechanisms (Gandhi and Abramov, 2012; Butterfield and Halliwell, 2019; Altanam et al., 2025). Polyphenols such as curcumin and resveratrol exert antioxidant effects by scavenging free radicals, enhancing endogenous antioxidant enzymes (SOD, catalase, glutathione peroxidase), and preserving mitochondrial integrity, thereby preventing oxidative neuronal injury (Butterfield and Halliwell, 2019).

Polyphenols activate Nrf-2/ARE pathway through two principal mechanisms: (a) polyphenols modify Keap1 (Kelch-like ECH-associated protein 1) releasing Nrf-2 to translocate to the nucleus, where it binds to the DNA’s antioxidant response element (ARE) within the promoter region and triggers the transcription of antioxidant defense genes, and (b) kinase-mediated signaling. Protein kinase C (PKC), mitogen-activated protein kinases (MAPKs), and PI3K/Akt are among the kinases that polyphenols can activate. These kinases phosphorylate Nrf-2 or its negative regulator, stabilizing Nrf-2 and promoting its nuclear translocation (Das et al., 2016; Zhou et al., 2019; Sidiropoulou et al., 2023).

Tea polyphenols have also been shown to reduce ROS and H_2_O_2_ levels to prevent mitochondrial dysfunction in H_2_O_2_-induced human neuroblastoma SH-SY5Y cells by initiating Keap1-Nrf-2 signaling and lowering H_2_O_2_-mediated cell death (Qi et al., 2017; Rahman et al., 2020). *Ginkgo biloba* extract is another well-known phytochemical source with potent antioxidant activity. Various biologically active compounds, such as trilactonic diterpenes, ginkgolides, and a number of flavonoids like quercetin, kaempferol, isorhamnetins, and proanthocyanidins, are present in the *Ginkgo biloba* extract (Singh et al., 2019). Free radical scavenging, ROS inhibition, metal chelation, and overexpression of antioxidant enzymes (SOD, glutathione reductase, and γ-glutamylcysteinyl synthase) have been shown by the standardized extract of *Ginkgo biloba*, named EGB761 (Plascencia-Villa and Perry, 2023).

On the other hand, caffeine and its catabolic metabolites xanthine and theobromine exhibit both pro-oxidative and antioxidant effects. Although pro-oxidative effects are attributed to its interaction with nucleic acids and transition metal ions, its antioxidant effects are attributed to its ability to scavenge free radicals and hydroxyl radicals (Plascencia-Villa and Perry, 2023). By protecting against oxidative stress and supporting mitochondrial function, phytochemicals help preserve synaptic integrity and neuronal function.

Anti-inflammatory actions: acute inflammation is a protective response against infection, toxic substances, and injury. However, chronic inflammation (neuroinflammation) arises when the balance between pro- and anti-inflammatory signaling becomes disrupted as observed in AD (Meraz-Ríos et al., 2013; Ferreira et al., 2014; Kinney et al., 2018). It has been demonstrated that phytochemicals such as quercetin, curcumin, resveratrol, and other flavonoids alter the gut-brain axis, affecting oxidative stress and neuroinflammation in the brain (Kim et al., 2024). Phytochemicals belonging to steroidal and terpenoid classes target a number of neuroinflammatory processes, such as the suppression of cytokine expression and microglial activation, which are linked to Aβ accumulation in AD. Additionally, these substances have anti-Aβ and anti-hyperphosphorylation qualities, which are essential for halting the advancement of AD (Kabir et al., 2021; Sharifi-Rad et al., 2022). Flavonoids like luteolin and quercetin suppress NF-κB signaling and reduce pro-inflammatory cytokine release (TNF-α, IL-1^+^), thereby restoring a neuroprotective microglial phenotype (Kwon and Koh, 2020). Recent evidence shows that flavonoids such as quercetin, rutin, anthocyanins and hesperetin attenuate astrocyte-driven neuroinflammation by supressing NF-κB, MAPK, TLR, and NLRP3 signaling while activating Nrf-2 and PI3K/Akt pathways. This dual modulation preserves astrocytic homeostasis, improves astrocyte-microglia communication, and enhances synaptic integrity and neurotrophic factors, ultimately promoting synaptic plasticity and cognitive resilience in neurodegeneration (Rao D. P. et al., 2026). Thus, phytochemicals, through this regulatory mechanism aid in preventing the chronic inflammation associated with AD pathology (Dey, 2019; Zhang et al., 2019; Kim et al., 2024). By suppressing neuroinflammatory signaling and modulating microglial activation, phytochemicals help protect synaptic structures and maintain neuronal communication.

Modulation of Aβ and tau pathology: phytochemicals, particularly polyphenols, prevent the formation of neurotoxic oligomers and Aβ plaques by inhibiting Aβ self-assembly. They reduce Aβ burden in the brain by enhancing the clearance of Aβ(1-42) monomers and converting toxic oligomers into non-toxic forms (Lakey-Beitia et al., 2015; Zheng et al., 2019). Several phytochemicals interfere with Aβ aggregation or promote its clearance. EGCG from the green tea remodels toxic Aβ fibrils into non-toxic aggregates, while curcumin binds Aβ plaques and inhibits their formation (Fernandes et al., 2021). Toxic Aβ oligomer production has also been modulated by polyphenols; Zheng et al. (2019) reported that resveratrol and EGCG transformed toxic Aβ oligomers into benign assemblies, whereas rosemarinic acid and curcumin directly disrupted peptide-peptide interactions to prevent oligomerization. Miyamae et al. (2011) investigated the prevention of Aβ protein aggregation in SH-SY5Y cells, where their findings demonstrated that caffeoylquinic acids (CQA), specifically 3,4,5-tri-O-caffeoylquinic acid and 4,5-di-O-caffeoylquinic acid, significantly decreased Aβ aggregation, indicating that the caffeoyl group in CQA is crucial for its inhibitory effect in the neuroblastoma model (Miyamae et al., 2011). Similarly, compounds such as ginsenosides and withanolides reduce tau hyperphosphorylation by regulating GSK-3β and MAPK pathways (Tancreda et al., 2025). Aβ oligomers are highly synaptotoxic and disrupt synaptic signaling by impairing synaptic receptor function and dendritic spine stability. By inhibiting Aβ aggregation and promoting its clearance, phytochemicals may therefore help preserve synaptic integrity.

Anticholinesterase mechanisms: the neurotransmitter acetylcholine (ACh) is essential for memory, learning, and synaptic plasticity. ACh depletion is a characteristic feature of AD (Deepa and Thomas, 2025). Many phytochemicals classified as alkaloids, polyphenols, flavonoids, and terpenoids have been shown to possess anti-inflammatory, antioxidant, and neuroprotective qualities in addition to the ability to block acetylcholinesterase (AChE) (Erdogan Orhan et al., 2011; Ahmed et al., 2013; Deepa and Thomas, 2025). Rehman et al. (2022) reported AChE inhibitory activity for compounds such as betanin and glycine betaine, with IC_50_ values of 1.271 and 1.203 μM, respectively. Similarly, strong AChE inhibition has been shown by extracts from 28 distinct plant species, with IC_50_ values ranging from 0.08 to 10.0 μg/mL (Deepa and Thomas, 2025). Among various phytochemicals reported in different studies, *Moringa oleifera* has been found to contain compounds such as quercetin and 4-O-caffeoylquinic acid that show strong AChE inhibitory activity along with notable antioxidant properties (Aljadaan et al., 2025).

Similarly, *Beta vulgaris* has been reported to possess bioactive constituents like myricetin and betanin, which exhibited potent AChE inhibition and encouraging binding affinities in experimental studies (Rehman et al., 2022). In the case of *Fernandoa adenophylla*, various studies have shown significant inhibitory activity against AChE and butyrylcholinesterase (BuChE), where peshawaraquinone was highlighted as one of the potent phytoconstituents, which indicates the importance of these plant-derived compounds in the modulation of neurodegenerative disorders (Maria et al., 2025). Collectively, these findings highlight the potential of plant-derived compounds as natural AChE inhibitors capable of improving cholinergic signaling and protecting synaptic function in neurodegenerative conditions.

Synaptic plasticity and neurotrophic support: impaired synaptic function is observed in early AD and can occur before neuronal loss. Phytochemicals such as resveratrol and bacosides from *Bacopa monnieri* have been shown to improve synaptic function by increasing the levels of brain-derived neurotrophic factor (BDNF) and activating cAMP response element-binding protein-mediated transcription, which helps in synaptic repair and cognitive resilience (Abdul Manap et al., 2019). Additionally, it has been demonstrated that flavonoids can pass through the BBB, modulating neuronal signaling and promoting synaptic plasticity by activating defense mechanisms against oxidative stress (Vauzour et al., 2008).

Phytochemicals support neuronal health by regulating neurotrophins, increasing levels of BDNF and NGF to promote neuronal growth, survival, and cognitive function (Venkatesan et al., 2015; Naoi et al., 2017). They also enhance synaptic plasticity via TrkB-mediated activation of PI3K/Akt and MAPK/ERK pathways, which drive synaptic protein expression, inhibit apoptosis, and improve neuronal resilience under neurodegenerative conditions (Davinelli et al., 2012; Naoi et al., 2017). Phytochemicals also represent a potentially promising multi-targeted treatment strategy for AD, which can modulate oxidative stress, neuroinflammation, Aβ and tau pathology, cholinergic dysfunction, and synaptic plasticity. The diverse actions of phytochemicals provide a clear advantage over traditional single-target compounds in addressing the complex pathology of AD (Kaur et al., 2017). Although phytochemicals face challenges related to their bioavailability and permeability through the BBB, improvements in formulation technology may provide a way forward.

(ii) Phytochemical modulation of synaptic plasticity and neuronal repair

Synaptic plasticity, which is a fundamental basis of learning and memory, has been found to occur mainly at the glutamatergic synapses, where presynaptically released glutamate acts on NMDARs, AMPARs, along with metabotropic glutamate receptors (mGluRs) to modulate synaptic strength (Kew and Kemp, 2005; Yoon et al., 2023). During LTP, intense synaptic activity relieves the Mg^2+^ block from the NMDA receptors, facilitating Ca^2 +^ influx that activates protein kinases, phosphorylates AMPA receptors, and drives transcription and structural reorganization of dendritic spines in the late phase (Sanhueza and Lisman, 2013; Buffington et al., 2014). Importantly, these LTP-regulating pathways are highly vulnerable to oxidative stress and neurodegeneration making them important targets for phytochemical intervention in AD.

Phytochemicals have also been recognized as significant modulators in synaptic plasticity and repair through their ability to control neurotrophic signaling as well as intracellular survival mechanisms. Compounds such as flavonoids, curcumin, quercetin, and EGCG, are well-established to possess neuroprotective properties due to their ability to reduce oxidative stress as well as neuroinflammation (Farooqui, 2012; Velmurugan et al., 2018). A key mechanism underlying these effects involve the regulation of BDNF, a critical neurotrophin involved in synaptic plasticity, and neuronal survival (Jeanneteau and Chao, 2013).

Phytochemicals have also been well-documented to upregulate the expression of BDNF and its receptor, tropomyosin receptor kinase B (TrkB), which in turn activates various downstream signaling cascades, such as ERK, Akt, mTOR, and CREB, among others. The activation of these signaling cascades have been implicated in the formation of dendritic spines, synaptic protein synthesis, as well as neuroprotection against stress-induced synaptic elimination in specific brain regions such as the hippocampus and prefrontal cortex (Singh et al., 2025; Yoon et al., 2023). In addition, BDNF-TrkB signaling further engages the PI3K/Akt and ERK1/2 pathways, which enhance neuronal survival by regulating Bcl-2 family proteins and suppressing apoptotic signaling (Lucken-Ardjomande and Martinou, 2005). Experimental evidence also suggests that some phytochemicals can directly affect neurogenesis and synaptic repair. For instance, curcumin has been shown to stimulate hippocampal neurogenesis and synaptic plasticity by enhancing the expression of BDNF and serotonin 1A receptor signaling pathways, thereby promoting neuronal proliferation in the dentate gyrus (Xu et al., 2007). Similarly, steroidal compounds found in medicinal plants also show promising neuroprotective effects. Spicatoside A, a steroidal saponin from *Liriope platyphylla*, has been reported to induce neurite outgrowth and increase NGF synthesis via TrkA-dependent activation of PI3K and ERK1/2 pathways (Hur et al., 2009; Kwon et al., 2014).

Plant-derived sterols such as β-sitosterol and stigmasterol, identified in *Ginkgo biloba*, have been reported to protect neurons from Aβ-induced toxicity by modulating inflammatory and apoptotic signaling pathways, including NF-κB, MAPK, and caspase-mediated mechanisms (Lee et al., 2018; Mishra et al., 2024). In addition to these compounds, Ginkgolide B, another bioactive constituent of *Ginkgo biloba*, has demonstrated significant neuroprotective effects *in vitro*, including restoration of antioxidant defense, enhancement of DNA repair, and preservation of mitochondrial integrity, highlighting its neuroprotective therapeutic potential in AD (Kaur et al., 2015, 2020; Gill et al., 2017). Furthermore, flavonoids obtained from *Passiflora* species have been reported to enhance neuronal activity and improve behavioral performance, suggesting a potential role in the regulation of synaptic plasticity (Barbosa et al., 2008; Xu et al., 2013).

Overall, the aforementioned studies suggest that phytochemicals have the capacity to influence synaptic plasticity and repair via a complex array of mechanisms, which include the modulation of glutamatergic transmission, activation of neurotrophic activity, and inhibition of apoptosis and inflammation. These plant-derived bioactive compounds may therefore sustain synaptic integrity, with possible applications for the prevention of synaptic pathology seen in neurodegenerative AD.

(iii) Phytochemical-derived therapeutics in clinical use for AD

The translational relevance of phytochemicals in AD is most directly demonstrated by plant-derived compounds that have successfully reached clinical use. Two FDA-approved drugs currently used for the symptomatic management of mild to moderate AD are directly derived from plant sources. Galantamine, an alkaloid originally isolated from *Galanthus nivalis* (common snowdrop, Amaryllidaceae family), functions as a dual-action agent. It is an AChE inhibitor and an allosteric potentiator of nicotinic acetylcholine receptors and represents the most established example of a phytochemical-based AD therapeutic (Ago et al., 2011; Heinrich, 2010). Rivastigmine, a semi-synthetic derivative structurally based on physostigmine from *Physostigma venenosum* (Calabar bean), is similarly approved as a dual inhibitor of both AChE and butyrylcholinesterase (BuChE), offering broader cholinergic coverage than galantamine alone (Kumar, 2006; Kandiah et al., 2017). Additionally, huperzine A, a potent reversible AChE inhibitor isolated from the Chinese club moss *Huperzia serrata*, demonstrates superior BBB penetration and has been widely used in clinical practice in China for the management of cognitive symptoms in AD (Wang et al., 2006; Friedli and Inestrosa, 2021). Among emerging candidates, curcumin and EGCG are currently under clinical investigation, with ongoing studies focusing on nanoparticle and liposomal delivery systems to overcome their inherently poor bioavailability and insufficient CNS penetration (Reddy et al., 2018; Fernandes et al., 2021). Despite these advances, it must be emphasized that no phytochemical has yet received global regulatory approval as a disease-modifying agent for AD, and rigorous, well-powered clinical trials remain essential to establish definitive therapeutic efficacy.

## 7 Summary and conclusion

Extracellular accumulation of Aβ plaques, intracellular hyperphosphorylated NFTs, and synaptic dysfunction are the early hallmarks of AD, driving memory decline and cognitive impairment. This review emphasizes how mitochondrial bioenergetics, cytoskeletal integrity, exosomal signaling, and immune responses like NETosis contribute to synaptic dysfunction in AD, highlighting the multitarget potential of phytochemicals in regulating various pathways to promote synaptic regeneration and repair. Mitochondrial dysfunction impairs synaptic integrity through defective OXPHOS, ATP depletion, and disrupted Ca^2+^ buffering. Mitochondrial complexes I and IV are frequently impaired in MCI and AD, which increases ROS levels that oxidize synaptic proteins, lipids and DNA. This interrupts LTP, leading to progressive loss of neuronal circuits. Mechanistically, elevated ROS activates p38 MAPK and JNK signaling that is associated with the degradation of synaptic proteins. Phytochemicals, including ferulic acid, curcumin, resveratrol and picrosides, also modulate Nrf-2/ARE pathway to restore redox balance and protect mitochondrial bioenergetics.

The cytoskeleton and mitochondria are functionally co-dependent in maintaining synaptic health. Actin filaments and microtubules coordinate the trafficking of healthy mitochondria to the synaptic terminal, where they can be recycled and continue to supply ATP for neurotransmission, or direct damaged mitochondria toward degradation pathways to limit oxidative stress. In addition to this trafficking role, the cytoskeleton itself represents another central axis of synaptic repair. Actin filaments generate forces for dendritic spine remodeling, while microtubules regulate receptor trafficking and vesicular transport. In AD, tau hyperphosphorylation (through GSK-3β and CDK5) destabilizes microtubules, and cofilin dysregulation disrupts actin turnover. This leads to a collapse of dendritic spines and disassembly of the synapse. Collectively, tau pathology operates at multiple levels including impairing axonal transport, corrupting cytoskeletal stability, trans-synaptic propagation via exosomes, and synergising with Aβ oligomers to amplify synaptic toxicity, underscoring its central role in AD pathogenesis. Stabilization of microtubules (e.g., epothilone D) or promotion of actin polymerization have shown promise in restoring the synaptic plasticity in preclinical models. The RhoA/Rac1/Cdc42 pathways are modulated by various phytochemicals such as curcumin, EGCG, ferulic acid and withanolide A to rescue cytoskeletal dynamics.

It is noteworthy that cytoskeletal proteins are not only present within cells but have also been reported as exosomal cargo with altered composition under AD conditions. In addition to proteins, lipids, and miRNAs that differentially modulate pathways, exosomes act as vectors of cytoskeletal elements, altering synaptic communication and thus establishing a further level of regulation in AD pathogenesis. Loss of exosomal miR-132/miR-124 leads to impaired synaptic plasticity, and astrocyte-derived exosomes enriched with complement proteins enhance synaptic pruning. On the other hand, exosomes loaded with BDNF, neurotrophic miRNA, or antioxidants could increase dendritic spine formation and synaptic resilience. Phytochemical compounds modulate exosome cargo composition and enhance the release of vesicles with neuroprotective cargo. Interestingly, exosomes harbor inflammatory mediators capable of modulating neutrophil and microglial activity linking immune dysregulation with vesicle-mediated signaling.

Immune dysregulation through NETosis also contributes to accelerated synaptic loss. Under these conditions, NETs containing DNA, histones and MPO, breach the BBB and activate microglia through TLR4 and NF-κB signaling pathways, thereby propagating oxidative damage and proteolytic degradation of synaptic components. NET formation is reduced upon inhibition of PAD4 (a key NETosis enzyme). Natural compounds such as quercetin have been reported to attenuate excessive NETosis and reduce neuroinflammation-driven synaptic injury.

**FIGURE 3:**
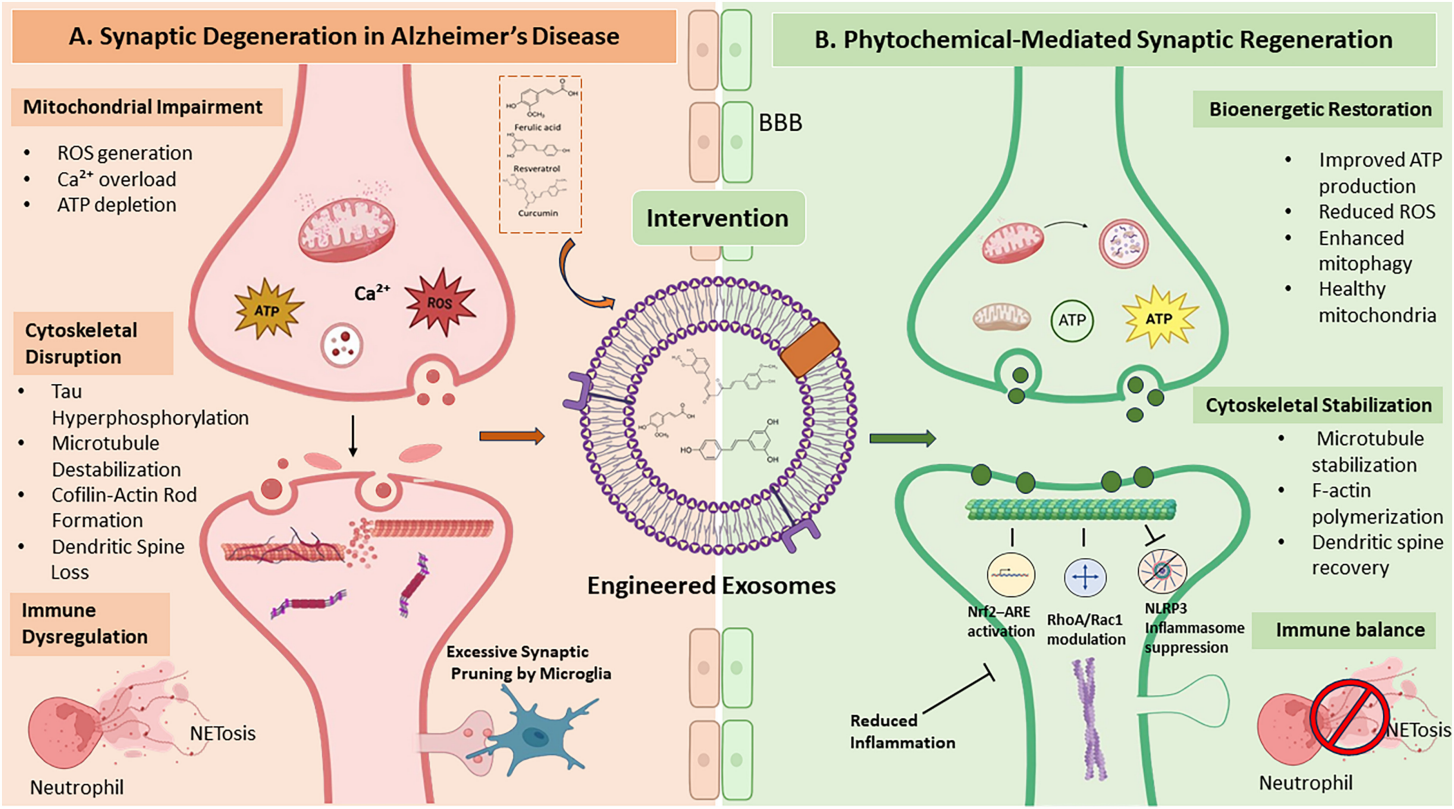
Schematic illustration of synaptic dysfunction in AD driven by mitochondrial impairment, cytoskeletal disruption, and immune dysregulation. **(A)** Synaptic degeneration in AD, characterised by mitochondrial impairment (ROS generation, Ca^2+^ overload, ATP depletion), cytoskeletal disruption (tau hyperphosphorylation, microtubule destabilisation, cofilin-actin rod formation, dendritic spine loss), and immune dysregulation (NETosis, excessive synaptic pruning by microglia). **(B)** Phytochemical-mediated synaptic regeneration via engineered exosomes crossing the BBB, activating protective pathways (Nrf-2-ARE, RhoA/Rac1 modulation) while suppressing NLRP3 inflammasome signalling, thereby restoring mitochondrial function, stabilising synaptic structure, promoting immune balance, and supporting synaptic repair.

Taken together, these pathways form a self-reinforcing cycle of synaptic injury in which mitochondrial dysfunction acts as the primary mechanistic driver. Mitochondrial ROS drives tau hyperphosphorylation and cytoskeletal collapse, which in turn impairs mitochondrial trafficking and axonal transport, thereby amplifying synaptic energy failure. This intracellular dysfunction is further propagated through exosomes carrying toxic cargo (Aβ, hyperphosphorylated tau, and inflammatory mediators), spreading pathology across synaptic network and sustaining neuroinflammation. This chronic inflammatory state promotes neutrophils recruitment and NETosis-associated BBB disruption by releasing proteases and pro-inflammatory signals thus exacerbates proteolytic damage at synaptic sites. The resulting increase in immune infiltration and metabolic stress further exacerbates mitochondrial dysfunction, thereby sustaining and amplifying the cycle of synaptic damage. In this context, the multi-targeted nature of phytochemicals is particularly relevant, as preclinical evidences suggest that they may act across several nodes of this network by supporting mitochondrial biogenesis, stabilizing cytoskeletal scaffolds, regulating exosomal miRNA packaging, and attenuating NETosis-driven inflammation. Innovative delivery systems such as exosome-based carriers and nanoparticles can further amplify their potency at the synaptic sites (Figure 3). Thus, future studies should employ biomarker-driven approaches to map the crosstalk among mitochondrial dysfunction, cytoskeleton disruption, exosomes signaling and immune dysregulation in AD. Combination strategies, such as utilizing phytochemicals in conjunction with exosomes and cytoskeletal modulators for targeted delivery are also promising. In conclusion, synaptic recovery in AD depends on multiple interconnected processes, and emerging key multitarget agents such as phytochemicals may help restore synaptic balance and improve cognitive resilience.

It is important to acknowledge that the majority of mechanistic insights discussed in this review are derived from *in vitro* studies and *in vivo* transgenic animal models. While these studies provide valuable understanding of disease pathways, direct clinical evidence linking these mechanisms to synaptic outcomes in human AD remains limited. Moreover, phytochemicals-based therapeutic strategies have yet to demonstrate consistent efficacy in clinical trials. Translation of these findings will require well-designed randomized controlled trials, validated and sensitive biomarker endpoints, and advanced delivery systems capable of achieving therapeutic concentrations at synaptic targets.

## 8 Future directions and therapeutic perspectives

Understanding synaptic damage and restoring synaptic functions in AD have gradually changed the way we approach therapy. Future strategies will need to focus on coordinated network repair rather than targeting pathways or a single molecule, as AD is a system failure, not the failure of a single target. Effective intervention therefore requires targeting multiple levels simultaneously. Since phytochemicals naturally have a variety of pharmacological properties, they present an excellent opportunity. Rather than modulating a single pathway, they can simultaneously regulate mitochondrial health, oxidative stress, neuroinflammation and synaptic signaling. For instance, Ferulic acid could restore energy status and promote mitochondrial biogenesis in preclinical studies, whereas quercetin could suppress excessive NETosis and inflammatory cascades. If formulated correctly, these molecules would stabilize the fragile synaptic microenvironment. However, limited bioavailability remains a significant obstacle, as many phytochemicals fail to achieve therapeutic effects due to low brain concentrations and BBB permeability. Thus, novel nanotechnologies and brain-targeted delivery systems, such as engineered exosomes, can cross the BBB and deliver drugs to weakened synapses. These targeted methods may guard against microglial over-pruning and Aβ-induced toxicity with fewer systemic side effects. Future therapeutic approaches should extend beyond neuronal targets to encompass the glymphatic-immune axis by exploring how phytochemicals may mediate clearance of both metabolic waste and neutrophil-derived traps that sustain chronic neuroinflammation. In particular, biomarkers in the MCI stage are essential for assessing synaptic status through exosomal profiling and enabling early corrective interventions. To that end, the emphasis in AD research is shifting away from passive neuroprotection towards proactive strategies to re-establish synaptic connectivity. As such, combination therapies involving cytoskeletal stabilizers and remodulators, various phytochemicals may have the potential to not only slow down the progression of disease, but also restore dendritic structures and cognition. Therefore, with appropriate delivery mechanisms, these combined approaches represent a promising therapeutic direction that requires systematic clinical validation, with the ultimate aim of mitigating disease progression and supporting synaptic resilience in AD.

Despite considerable promise, the clinical translation of phytochemical-based strategies remain constrained by several factors. Most preclinical studies employ supraphysiological concentrations that may not be achievable in the human brain. Additionally, multifactorial nature of AD often limits the direct translation of findings from animal models to human trials, as reflected in the limited success of several single-targeted approaches. Although the polypharmacological properties of phytochemicals are conceptually advantageous, they also introduce challenges in dose standardization, safety evaluation, and regulatory approval. Beyond pharmacokinetic challenges, the safety of phytochemicals at therapeutic doses requires careful consideration. Although generally regarded as safe, high-dose or prolonged use may cause adverse effects, including gastrointestinal disturbances (curcumin), pro-oxidant activity at high concentrations (resveratrol), and hepatotoxicity (EGCG). Phytochemicals may also interact with medications, for example, resveratrol and quercetin inhibit CYP450 enzymes (CYP3A4 & CYP2C9), potentially altering the metabolism of drugs such as warfarin, statins, and cholinesterase inhibitors, while *Ginkgo biloba* may increase bleeding risk due to its anticoagulant effects. Addressing these challenges through pharmacokinetic optimisation, validated biomarker endpoints, and adaptive clinical trial designs will be essential for translating preclinical promise into clinical benefit.
